# Psychometric Challenges in the Measurement of Constructs Underlying Criminal Responsibility in Children and Young Adults: A Cross-Sectional Study

**DOI:** 10.3389/fpsyg.2021.781669

**Published:** 2022-01-13

**Authors:** Yuxi Shang, Yumiao Fu, Beibei Ma, Li Wang, Dexin Wang

**Affiliations:** ^1^School of Law, Shandong Normal University, Jinan, China; ^2^Master of Law Education Centre, East China University of Political Science and Law, Shanghai, China; ^3^School of Psychology, Shandong Normal University, Jinan, China

**Keywords:** juvenile, minimum age of criminal responsibility, dialectical thinking, self-control, empathy

## Abstract

At present, many countries have lowered the minimum age of criminal responsibility to deal with the trend of juvenile crime. In practical terms, whether countries advocate for lowering the age of criminal responsibility along with early puberty, or regulating the minimum age of juvenile criminal responsibility through their policies, their deep-rooted hypothesis is that age is tied to adolescents’ psychological growth, and, with the rise in age, the capacity for dialectical thinking, self-control, and empathy gradually improves. With this study, we aimed to test whether this hypothesis is valid. The participants were 3,208 students from junior high school, senior high school, and freshman in the S province of the People’s Republic of China (PRC). We subjected the gathered materials to independent-samples *t*-tests, one-way analysis of variance (ANOVA), linear regression analysis, and Bonferroni *post hoc* test. The influence of the age variable upon dialectical thinking, self-control, and empathy was significant (*p* = 0.002, *p* = 0.000, *p* = 0.072), but only empathy was positively correlated with age variable (*B* = 0.032); dialectical thinking ability (*B* = −0.057), and self-control ability (*B* = −0.212) were negatively correlated with the age variable. Bonferroni *post hoc* test confirmed these findings. Therefore, we concluded the following: (1) Juvenile criminal responsibility, based on the capacity for dialectical thinking, self-control, and empathy, is not positively correlated with age. (2) Age is not the only basis on which to judge a juvenile’s criminal responsibility. (3) More research that directly links age differences in brain structure and function to age differences in legally relevant capacities and capabilities(e.g., dialectical thinking, self-control, and empathy) is needed. (4) Political countries should appropriately raise the minimum age of criminal responsibility and adopt the doli incapax principle in the judicial process.

## Introduction

On October 24, 2019, a 13-year-old boy in Dalian in the People’s Republic of China (PRC) killed a 10-year-old girl and dumped her body in his home in a brutal manner (Xue et al., 2019). Similarly, on the other side of the world, in November of 2015, news that an 8-year-old child had been held in custody for viciously attacking and killing a 1-year-old child in the United States state of Alabama circulated in the media (Crofts, 2016). With the rapid spread of media reports and communications, an increasing number of malignant incidents committed by young minors have come into the public view.

Faced with the exposure of many younger malignant criminal cases, some countries and regions have chosen to implement strict laws for juvenile offenders; that is, to lower the age of criminal responsibility, so as to try to achieve the goal of social defense by cracking down on juvenile delinquency. Like America in the 1980s, with rising juvenile crime rates and media attention (Butts and Mitchell, 2000; Cook and Laub, 2002), some nations have lowered the age threshold for sending a teenager into the criminal justice system (some have lowered it to 12 years old), ushering in a “Hard Age” of juvenile justice in United States (Fowler and Kurlychek, 2018). Recently, facing the rising trend of juvenile delinquency, the South Korean government is trying to lower the minimum age of criminal responsibility from 14 to 13 (Hong, 2020). On December 22, 2020, at the 24th session of the 13th Standing Committee of the National People’s Congress of the PRC, an amendment (XI) was made to the criminal law to lower the minimum age of criminal responsibility for the murder and aggravated assault that causes death from 14 to 12 years old (NPC, 2020).

Is there a scientific basis for lowering the minimum age of criminal responsibility in adolescents? In addition to curbing the rise of juvenile crime rates and eliminating the public’s fear and risk of victimization by juvenile delinquents (Blumstein, 1995; Baker et al., 2016; Sickmund et al., 2017), proponents point to the “early onset of [the] concept of right and wrong and understanding of the meaning of one’s behavior as socioeconomic development and online life spread” (Rosenfield et al., 2000; O’Brien and Fitz-Gibbon, 2017). However, there are many objections, such as exposing young people to the criminal justice system at an early age does not produce a good preventive effect, but instead leads to an increase in the rate of juvenile recidivism (Doreleijers and Fokkens, 2010; Casey, 2014; O’Brien and Fitz-Gibbon, 2017). Unfortunately, these arguments rest on theoretical discussions or indirect proof (Newton and Bussey, 2012). It is well known that the basis of criminal responsibility in juveniles is the capacity for criminal responsibility; that is, appreciation and self-control (Zhang, 1994; Snyman, 2008; Elliott, 2011). Appreciation is the actor’s capacity to distinguish the meaning, nature, function, and consequence of their behavior in criminal law (Snyman, 2008; Goldson, 2013). Self-control is the ability to moderate one’s actions and to act in accordance with the law (Goldson, 2013). Whether countries advocate for lowering the age of criminal responsibility along with early puberty or regulating the minimum age of juvenile criminal responsibility through their policies, one deep-rooted hypothesis is:

H_1_: Age is related to adolescents’ psychological growth, and with increasing age, the capacity for appreciation and self-control gradually improves.

Our main objective was to test the validity of such a hypothesis using quantitative methods. Combining the basis of juvenile criminal responsibility (appreciation and self-control), we quantified juvenile criminal responsibility using three psychological indices:

(1) *The index of dialectical thinking*. Dialectical thinking skills enable adolescents to see the world objectively, observe events, and deal with problems in all aspects (Inhelder and Piaget, 1958; Nisbett et al., 2001; Cheng, 2009; Boucher, 2011). Moreover, the development of dialectical thinking ability can effectively reduce people’s aggressive behavior (Zhang et al., 2011). In contrast, adolescents with inadequate development of dialectical thinking skills are prone to attribution bias and risky behaviors (Crick and Dodge, 1994).
(2) *The index of self-control*. Self-control is the capacity to suppress inappropriate emotions and behaviors, and to replace them with appropriate ones (Casey, 2014). Low self-control is often the root of problematic behaviors, like poor interpersonal relationships, job prospects, health, and especially of involvement in antisocial and criminal conduct (Walters, 2016). This is consistent with Gottfredson and Hirschi’s (1990) assertion that low self-control is a major cause of crime.

Importantly, some countries have set a lower age of criminal responsibility for violent crimes. For example, the Russian Criminal Code (1996) stipulates that a person can be held criminally liable for any offense committed from the age of 16, and a child aged 14 or older can be held criminally liable for a number of serious violent crimes such as willful murder, rape, and robbery (Criminal Code, Article 20). In Ireland, children under 12 can generally not be prosecuted, but children over the age of 10 can be prosecuted for certain crimes such as murder, manslaughter, rape, or aggravated sexual assault (Crofts, 2016).

Psychological research has found that the development of empathy has an impact on juvenile delinquency (Narvey et al., 2021). For example, Miller and Eisenberg’s (1988) study found a significant negative correlation between empathy and aggressive behavior, especially in adolescence. Empathy is the cognitive ability to experience and understand the emotions of others (Jolliffe and Farrington, 2004). Empathy is present in the early years of life, and infants have high levels of emotional empathy (Haviland and Lelwica, 1987). Brink et al. (2011) showed increased activation in the medial orbitofrontal cortex, left inferior frontal gyrus, and left dorsolateral prefrontal cortex in a story task that elicited emotional empathy. Low empathy is often associated with aggression and criminality. Research on the relationship between empathy and types of crimes found a significant correlation between sexual and violent crimes and low empathy (Harpur et al., 1988; Jolliffe and Farrington, 2004, 2006a,b). Likewise, high empathy reduces violence and aggression (Broidy et al., 2003).

(3) Hence, in addition to the dialectical thinking and self-control indices, we also regard the index of *empathy* as a standard with which to measure the level of juvenile criminal responsibility. Our previous argument for H_1_ could be modified to H_2_:

H_2_: Age is related to adolescents’ psychological growth; the capacity for dialectical thinking, self-control, and empathy is positively correlated with age, and a developmental (or stable) level of empathy occurs earlier than the level of dialectical thinking and self-control.

## The Current Study

Regarding the importance of age in the criminal responsibility system, it is the threshold that determines whether a juvenile will enter the criminal justice system (Crofts, 2016). Different countries have different age levels due to distinct historical traditions and cultures (Pillay, 2019), and there is no consensus on which age level is appropriate. In existing research, the area of the relationship between age and criminal responsibility is understudied. Therefore, we aimed to explore the connection between age and criminal responsibility; more specifically, whether juveniles’ criminal responsibility ability is positively correlated with age, and whether they tend to have the capacity for adult criminal responsibility at a certain age. It is important to test the deep-rooted belief that age is the criterion for determining adolescents’ criminal responsibility; with the development of society and the maturity of teenagers, the minimum age of criminal responsibility can be adjusted. We used quantitative analysis. First, through questionnaires, we measured adolescents’ capacity for dialectical thinking, self-control, and empathy in order to establish a propensity for violent crime. Second, under the control of demographic variables such as academic achievement, parental occupation, and socioeconomic status, we analyzed the relationship between the three indices and adolescents’ age. Finally, we attempted to address the following questions:

• Is adolescents’ criminal responsibility (the capacity for dialectical thinking, self-control, and empathy) positively correlated with age?
  • If so, will adolescents’ capacity for dialectical thinking, self-control, and empathy become more stable (mature) or more adult-like at some point in their lives?
  • If not, what does the developmental trend of young people’s capacity for dialectical thinking, self-control, and empathy look like?
• Does the minimum age of the criminal responsibility system need to be reformed? If so, how?

## Materials and Methods

### Data

The first sample consisted of students from grades 6 through 12(ages from 10 to 22) in S County, in S province of the PRC. S County is located in China’s eastern coastal region, where young students have access to more advanced educational methods and technologies, but the level of economic growth is in the middle compared to the rest of the country (in 2020, S county’s per capita disposable income was [PCDI] = [¥17,046, ¥30,933]; the PRC’s per capita disposable income was [PCDI] = [¥15,204, ¥43,834]). To improve the representativeness of the sample, we used a whole group random sampling method to select 2,800 participants out of 27,031 students from urban and rural areas in S County. As we chose the high schools through a unified examination from the junior high schools in urban and rural areas, we did not distinguish between rural and urban schools. We divided the samples into a primary school group, a junior high school group, and a senior high school group. The primary school group only included pre-primary students (grade 6); we randomly selected 200 out of 2,659 pre-primary students from urban primary schools, and 200 out of 2,637 pre-primary students from rural primary schools, totaling 400 students. The junior high school group included students from grades 7 through 9. We randomly selected 200 urban school students and 200 rural secondary school students from each grade, totaling 1,200 students (the total number of students in each grade is 4,032, 3,940, 3,354 respectively). The senior high school group included students from grades 10 through 12. We randomly selected 400 students from each grade, totaling 1,200 students (the total number of students in each grade is 3,696, 3,477, 3,236, respectively). In all, we selected 2,800 samples, each of whom completed three questionnaires. We distributed a total of 8,400 paper-based questionnaires and collected 8,379, with a recovery rate of 99.75%. We found a small number of students aged 18 and over after the initial sample selection. We also conducted a second supplementary selection. From S University in S province, we chose 415 freshmen to fill out a questionnaire survey with a recovery rate of 100%; the final sample size was 3,208(ages from 11 to 19), the total recovery rate of 99.76%.

All procedures involving human participants in this study have been approved by the ethical standards of the Academic Board of Shandong Normal University. Participation was voluntary and anonymous, based on written informed consent and the right to withdraw participation at any time. We also obtained their guardians’ consent for minors under age 18. To comply with the requirements of COVID-19 prevention and control, we could not personally enter the campus to hand out and administer the questionnaires, so they were handed out by school teachers who had received professional training. To a certain extent, this can ensure the legality and validity of the experimental data source.

We first preprocessed the data, using SPSS AU to screen out 35 invalid samples, and employing SPSS software to eliminate extreme questionnaire scores in each age group, excluding 29 samples. Due to the small number of samples aged 10 and over the age of 20, we excluded 58 samples from these two age groups, leaving 3,086 samples and 9,258 valid questionnaires. The preliminary analysis showed that the final sample was 47.4% male and 52.6% female; 70.3% of the respondents came from rural families and 29.7% from urban families. Regarding parents’ education level, 77.9% of students had fathers, and 82.8% had mothers, who had graduated from junior high school and below. Meanwhile, 22.1% of students had fathers, and 17.2% of students had mothers, who had completed senior high school or above. This is in line with the education levels of parents of middle school students in the PRC (Ji et al., 2018). The respondents ranged in age from 11 to 19, and the distribution proportion of respondents in different age stages is shown in Table 1.

**TABLE 1:** The age distribution of the respondents.

Age (*M* = 15.1; SD = 2.3)	Proportion (%)
11	5.2
12	11.8
13	12.8
14	12.9
15	12.9
16	12.0
17	11.8
18	15.1
19	5.5

*N = 636. M, mean; SD, standard deviation.*

### Measures

#### Brief-Dialectical Self Scale

In 2016, Spencer-Rodgers et al. (2004) developed a self-report questionnaire called the Dialectical Self Scale (DSS). The scale has been translated into many languages. We adopted the brief Chinese version (B-DSS), α = 0.71, with 14 items. The scale has been shown to have good validity in previous studies (Hamamura et al., 2008; Spencer-Rodgers et al., 2008, 2010; Liu et al., 2013). The scale contains a 7-point scoring system from “very different” to “very much agree,” and encompasses the three dimensions of conflict tolerance, cognitive change, and behavioral change, thereby reflecting people’s dialectical thinking level. The higher the scale score, the higher the dialectical thinking level.

#### Self-Control Ability of Middle School Students Questionnaire

This questionnaire was developed by Wang and Lu (2004), scholars of the PRC. Adolescents’ capacity for self-control is mainly reflected in three dimensions: emotional self-control, behavioral self-control, and thinking self-control. The split-half reliability is 0.856 (Wang and Lu, 2004). The scale has been shown to have good validity in previous studies (Wang and Lu, 2004; Feng et al., 2021; Tan, 2021). The questionnaire has a total of 36 items, including 10 forward-scoring questions and 26 reverse-scoring questions. Each item uses a 5-point scoring system, ranging from “totally disagree” to “totally agree.” The higher the score, the stronger the self-control.

#### Basic Empathy Scale

There are many tools for measuring empathy, such as the widely used Interpersonal Response Indicator (IRI) in the PRC, but these scales have been questioned for confusing empathy with sympathy. Hence, for this study, we used the Basic Empathy Scale (BES; Darrick and David, 2006). The BES is divided into two dimensions: emotional and cognitive empathy. The scale contains 20 items, including 8 items for negative scoring and 12 items for positive scoring; the higher the score, the greater the respondent’s empathy. Li et al. (2011) tested the structure of theoretical factors and the reliability and validity of the BES in the youth population of the PRC. They found that the BES met the relevant requirements of psychometrics (α = 0.777). The scale has been shown to have good validity in previous studies (Darrick and David, 2006; Li et al., 2011).

### Plan of Analysis

We employed SPSS 19.0 to analyze the results. Before doing so, we calculated the scores of the B-DSS, Self-Control Ability of Middle School Students Questionnaire (SAMSSQ), and BES (this score is the average score of each item on the scale). The missing values in the scores are filled in by the mean of the scores in the sample’s age group (Jin and Yu, 2015). After that, we employed the independent-samples *t*-tests and one-way ANOVA to gauge the influence of demographic variables on the capacity for dialectical thinking, self-control, and empathy. After controlling for the demographic variables, we observed the relationship between dialectical thinking, self-control, empathy, and age. Second, using two-variable correlation analysis, we explored whether it was necessary to carry out multivariate analysis of variance (MANOVA) on each dimension of the B-DSS, SAMSSQ, and BES. We used linear regression to derive the explanatory power of age for dialectical thinking, self-control, and empathy. Third, we employed the Bonferroni *post hoc* test, and further scrutinized the differences across ages in terms of dialectical thinking, self-control, and empathy.

## Results

### Covariance Analysis

Table 2 shows the variance analysis of demographic information using the independent samples *t*-tests and one-way ANOVA. We employed independent samples *t*-tests for the two categorical variables (including gender, family location, and class member status) and we used one-way ANOVA for three or more variables (including grade, achievement ranking, father’s level of education, mother’s level of education, family income). The data in Table 2 reveal that, in addition to age, other factors affected the B-DSS, SAMSSQ, and BES scores: The differences among the B-DSS, SAMSSQ, and BES scores across different grades were statistically significant. Gender only had an effect on the SAMSSQ and BES scores. Grade, whether the student’s family was living in an urban or rural area, and whether the student was part of a class committee had an impact on the SAMSSQ and BES scores. However, parents’ education and family economic level only affected the SAMSSQ scores.

**TABLE 2:** Variance analysis of the demographic variables and the scores for the three kinds of abilities.

Variables	B-DSS	SAMSSQ	BES
	T/F	T/F	T/F
Age	3.381*	66.349**	1.267
Gender	0.567	−2.985**	−11.611**
Achievement ranking	0.701	94.734**	8.843**
Family location	–0.151	5.848**	2.275*
Father’s education level	0.635	8.845**	1.446
Mother’s education level	0.424	10.832**	1.098
Family income	3.131*	55.858**	2.873
Class member status	0.409	34.407**	−5.100**

*Gender coded as (1 = male, 2 = female).*

*p < 0.05.

**p < 0.01.

Other factors besides age may influence judgments about the relationship between age and dialectical thinking, self-control, and empathy. Table 3 controls for these relevant demographic variables, revealing the scores of the B-DSS, SAMSSQ, and BES of each age group. Covariance analysis indicated that the difference between age and the B-DSS scores was statistically significant (*F* = 2.646, *p* = 0.007). Likewise, the difference between age and the self-control scores was statistically significant (*F* = 28.788, *p* = 0.000). The difference between age and empathy was not statistically significant (*F* = 1.086, *p* = 0.370).

**TABLE 3:** Descriptive analysis of age and the scores of the three kinds of abilities.

Scale	*B-DSS*		*SAMSSQ*		*BES*	
Age	*M*	SD	*M*	SD	*M*	SD
11	4.376	0.038	3.859	0.041	3.653	0.036
12	4.397	0.025	3.747	0.027	3.586	0.024
13	4.355	0.024	3.558	0.025	3.600	0.023
14	4.369	0.024	3.340	0.025	3.613	0.022
15	4.447	0.024	3.249	0.025	3.651	0.022
16	4.357	0.025	3.260	0.026	3.637	0.023
17	4.349	0.025	3.279	0.027	3.667	0.024
18	4.284	0.022	3.396	0.024	3.646	0.021
19	4.323	0.037	3.484	0.039	3.615	0.035

*N = 636. M, mean; SD, standard deviation.*

### Linear Regression Analysis

Age and the scores of the three abilities are numerical variables. The normalized residuals of the dependent variables (the three scales’ scores) followed a normal distribution, which confirmed that our study met the requirements of the linear regression analysis. We test a linear regression model that explored the effects of age on the capacity for dialectical thinking, self-control, and empathy after controlling for the demographic variables, and mainly tested the interpretation level and direction of age for the three abilities. The outcomes of linear regression showed that age was correlated with the capacity for dialectical thinking (*p* = 0.002; 95%CI = [−0.268, −0.061]) and self-control (*p* = 0.000; 95%CI = [−2.137, −1.540]), which is consistent with the results of the variance analysis of the questionnaire scores and the demographic variables in Table 2. The linear regression also indicated a correlation between age and the capacity for empathy (*p* = 0.072; 95%CI = [−0.011, 0.969], although *p* > 0.050, but p was within the range of acceptability), which was different from the outcomes of one-way ANOVA (*p* = 0.370). Besides, the linear regression data showed that the explanatory power and correlation direction of age to the three abilities were different. Age was explained by 0.4% of the variance in dialectical thinking (Nagelkerke’s *R*^2^), which pointed to a negative correlation (Beta = −0.057). Age accounted for 12.9% of the variance in self-control (Nagelkerke’s *R*^2^), with a negative correlation (Beta = −0.212). Age accounted for 5.2% of the variance in empathy (Nagelkerke’s *R*^2^), with a positive correlation (Beta = 0.032).

After completing the above linear regression analysis, we needed to explain why we did not conduct a multivariate analysis of variance for dialectical thinking, self-control, and empathy, and why we did not analyze the dimensions of the three scales. The two-variable correlation analysis, shown by Table 4, suggests that dialectical thinking, self-control, and empathy are correlated, but their Pearson’s product-moment correlation coefficients were all less than 0.3, which indicates that they were independent. After scoring the same type of questionnaire on different dimensions, data analysis demonstrated that the Pearson’s product-moment correlation coefficients for conflict tolerance, cognitive change, and behavioral change (three dimensions) on the B-DSS were all less than 0.3. The Pearson’s product-moment correlation coefficients for emotional self-control, behavioral self-control, and thinking self-control (three dimensions) on the SAMSSQ were all higher than 0.6, and the Pearson’s product-moment correlation coefficients for emotional empathy and cognitive empathy (two dimensions) on the BES were lower than 0.3.

**TABLE 4:** Two-variable correlation analysis

Variables	Dialectical thinking	Self-control	Empathy
Dialectical thinking	–		
Self-control	−0.170**	–	
Empathy	0.123**	0.037*	–

*p < 0.05.

**p < 0.01.

### Bonferroni *post hoc* Test

**FIGURE 1:**
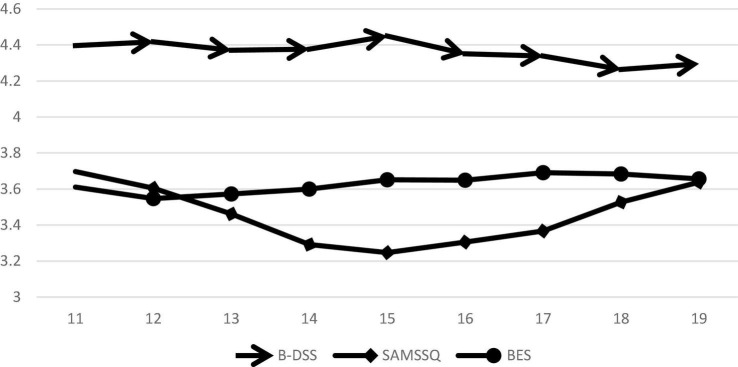
Trend chart of the scores for the three abilities along with age.

The ANOVA showed that age was correlated with the capacity for dialectical thinking, self-control, and empathy. The linear regression analysis explains the degree and direction of the interpretation of age for the three abilities as a whole. The differences in these three abilities in each age group have not been fully revealed. The Bonferroni *post hoc* test (Supplementary Appendix A) was able to specifically compare the three abilities at different ages. Figure 1 is based on the mean scores of the capacity for dialectical thinking, self-control, and empathy at different ages. Figure 1 and Supplementary Appendix A present the following: (1) The B-DSS scores were highest at age 15 (up: 11–15) and then fluctuated up and down (down: 15–18; up: 18–19). However, the dialectical thinking score of the 18-year-old group was lower than that of the 15-year-old group, and there was a significant difference (*p* = 0.000). (2) The self-control scores showed a more obvious, U-shaped trend with increasing age; the scores of students aged 11–15 decreased, those of 15–19 years old increased, and those of 14–16 were the lowest. The score of 11-year-olds was higher than that of 18- and 19-year-olds (*p* = 0.000), and the score of 12-year-olds was higher than that of 18- and 19-year-olds (*p* = 0.000). (3) The correlation between age and empathy was acceptable (*p* = 0.072). Overall, the BES scores indicate an increasing trend with age (12–18 years). Regarding the rising curve (BES), 16-year-old individuals had a slightly different score (less than 15-year-olds, but still more than 14-year-olds). However, the results of the Bonferroni *post hoc* test showed no significant difference in BES scores between different age groups (Supplementary Appendix A).

## Discussion

The age at which minors can be punished is controversial in different political countries (O’Brien and Fitz-Gibbon, 2017; Noroozi et al., 2018; Brown and Charles, 2019; Pillay, 2019; Schmidt et al., 2020). We employed a quantitative analysis of research methods, focusing on whether age can be used as a basis for measuring criminal responsibility, while also paying attention to the minimum age of criminal responsibility for violent crimes. The results of correlation tests showed that the influence of the age variable upon dialectical thinking, self-control, and empathy was significant, but only empathy was positively correlated with age variables (but the results of the Bonferroni *post hoc* test showed no significant difference in BES scores between different age groups). Dialectical thinking ability and self-control ability were negatively correlated with the age variable. This basically disproves the underlying hypothesis that countries should set a minimum age of criminal responsibility for juveniles, and indicates that the capacity for appreciation and self-control is positively correlated with age (H_2_). These results will be explained next.

### Dialectical Thinking Ability

We found that dialectical thinking does not increase with age; adolescents’ dialectical thinking is in a constant state of development until the age of 15, reaches a maximum then, and afterward declines. Next, it shows an upward trend after the age of 18. This finding is consistent with previous research on the current state of dialectical thinking development in middle school students (Lin and Qingan, 2005; Zhang, 2014). From age 11 (12) to age 17 (18) is the period when the mode of thinking transitions from the stage of formal operation to the stage of dialectical thinking (Inhelder and Piaget, 1958; Lin and Qingan, 2005). Generally speaking, with the increase of age, the dialectical thinking ability of minors is gradually increasing, which is not consistent with our conclusion (this trend does not begin until the age of 18). In other words, the development of adolescents’ dialectical thinking is not only unbalanced, but also possibly delayed. This may be related to the changing environment in which we live. Currently, adolescents are mired in a changing and fast world, especially with the advancements of smartphones and online games, which makes the thinking of teenagers become more simple and flat, and they gradually lose their interest in deep thinking of things (Ye and Li, 2005; Zheng, 2018).

On the other hand, dialectical thinking arises in the post-formal operations stage of Piaget’s cognitive developmental phases (Nisbett et al., 2001), in which individuals are able to see things and deal with problems in a holistic, connected, and developmental manner; this stage is also the last and highest of Piaget’s series of cognitive phases (Inhelder and Piaget, 1958). Lin and Qingan (2005) proposed that the dialectical thinking development of teenagers is the foundation laid by the knowledge learning in middle school. However, because it is the advanced stage of the development of cognition or thinking, the lag of development is inevitable.

In addition, emerging adults are in the process of identity exploration, during which they perceive themselves as neither teenagers nor adults and are unable to take responsibility and make decisions on their own, thus there is a lag in cognitive development in emerging adults (Arnett, 2000; Zheng, 2018; Kang, 2020). Of course, it should be noted that the development of dialectical thinking of adolescents after the age of 18 (early youth) needs to be further verified due to the limited data of subjects after the age of 18.

### Self-Control

Self-control ability showed a U-shaped trend, reaching a minimum at approximately 15 years of age and rising again afterward. This is consistent with the findings of Wang and Lu, who created the SAMSSQ (Wang and Lu, 2004). The reason for these outcomes is that adolescents enter puberty at approximately 15 years old, a period of physical, psychological, and hormonal changes (Choudhury et al., 2008). With the increase of age, adolescents become more independent and want to get rid of the restrictions of adults, both dependent and rebellious to adults, and sometimes appear out of control (Wang and Lu, 2004). Emotionally, they sometimes appear unstable, and this imbalance in psychological development makes their self-control no longer as good as before.

Related brain imaging evidence suggests that the maturation and development of relevant tissues in the brain during adolescence do not always increase linearly, but also present a non-linear curve of development (Gogtay et al., 2004; Toga et al., 2006). For example, frontal cortex activity increases between childhood and adolescence, and decreases between adolescence and adulthood (Choudhury et al., 2008).

Further, the influence of the social environment is particularly evident during puberty (Steinberg et al., 2008; Somerville, 2013; Blakemore and Mills, 2014), and adolescents undergoing puberty are more susceptible to peer influences (Guyer et al., 2012). The presence of peers made teens more likely to engage in risky behavior. And teens exhibited relatively greater activation in the ventral striatum and orbitofrontal cortex when their peers were observing them than when they were alone (Chein et al., 2011). Steinberg and Monahan (2007) interpreted these findings to mean that peers elicit a higher motivational state, which then activates the individual’s awareness, leading to a decrease in self-control.

### Empathy

Regarding the development of empathy, there seems to be an upward trend from visual observation. However, the variability between age and the acquisition of empathy was not very significant (linear analysis: *p* = 0.072, one-way ANOVA: *p* = 0.370), the results of the Bonferroni *post hoc* test also verified this result. The results are consistent with the dual processing model theory of empathic lifelong development (Huang and Su, 2010; Wang et al., 2021). This model suggests that the developmental trajectory of individual emotional empathy follows a U-shaped curve; its intensity remains relatively stable between adolescence and adulthood and then gradually increases (Liu and Cui, 2020). According to the results of this study, there is a linear trend in the development of adolescent empathy, which depends on a certain neuroscience basis (Wang et al., 2021). The maturation of the empathy response is closely related to the maturation of the prefrontal cortex (PFC), and adolescence is a critical period for individuals to reach the level of prefrontal cortex maturation (Yang et al., 2017). Decety et al. (2008) found that the ventromedial prefrontal cortex, which is strongly associated with cognitive empathy, becomes more active with age from childhood through functional magnetic resonance imaging.

Other researchers have found that the brain regions related to cognitive empathy such as the right temporo-parietal junction area and the left inferior frontal gyrus were significantly activated, while the brain regions related to emotional empathy did not show significant activation when someone else suffered a loss (Schwenck et al., 2017). This reflected the maturation of individual empathy, the stability of emotional empathy, and the development of cognitive empathy (Kunzmann et al., 2018). The development of empathy has an impact on juvenile delinquency (Narvey et al., 2021), which is also the reason why many political countries set a lower age of criminal responsibility for violent crimes. However, this kind of action needs further discussion, because the setting of criminal responsibility for minors is one that requires the simultaneous consideration of dialectical thinking, self-control, empathy.

## Limitations and Future Studies

Although we reached significant conclusions regarding the relationship between age and criminal responsibility, our study also faced some limitations.

First, although supplementary analyses and the control of covariates enhanced the explanatory power, the current study is essentially just a cross-sectional study, meaning that it cannot serve to answer the question about the longitudinal association between age and the capacity for dialectical thinking, self-control, and empathy. Future longitudinal investigations (e.g., a follow-up survey can be conducted with a group of eight-year-olds to explore the trends of these three abilities from 8 to 25 years old) and cross-lagged analyses would help to address these limitations.

Second, because all of our data came from student self-assessment, although we emphasized the authenticity and confidentiality of questionnaire responses during the student response process, issues such as social desirability and student concerns may have influenced the data collected on students’ dialectical thinking skills, self-control, and empathy, and future research could evaluate the above three skills in terms of peers, teachers and parents.

Third, there were limitations regarding the participants. As we found in the analysis of demographic variables, SES, including the role of juvenile parents and place of residence, is correlated with the three abilities of minors. However, we only sampled the population in province S, and although province S, as the second most populous province in the PRC, is highly representative, its representativeness to highly developed economic regions such as Beijing and Shanghai has yet to be verified due to the limitations of its economic growth. Therefore, subsequent studies should sample from populations nationwide to explore whether there are differences in dialectical thinking, self-control, and empathy among adolescents from different regions, ethnic groups and SES. The development of juveniles varies greatly from country to country due to differences in history, culture, level of economic development, and geography, so our conclusions cannot be universally applied to all nations (Yin and Du, 2014). Each state should choose a minimum age of criminal responsibility according to the developmental situation of its juveniles.

Last, in our study, age as the basis of criminal responsibility is challenged only from the aspect of psychology, which requires a more scientific basis, such as evidence from neuroscience and physiology (Carroll, 2015; Steinberg, 2017). For example, J.D.T., a 10-year-old boy sexually assaulted a 5-year-old boy, was controversially charged by the federal government, as J.D.T. had an undetectable level of testosterone in his bloodstream (Hamilton and Turner, 2015). As we mentioned in the discussion section, current explanations of adolescent development are based more on brain science. More research that directly links age differences in brain structure and function to age differences in legally relevant capacities and capabilitie (e.g., dialectical thinking, self-control, and empathy) is needed. In light of recent developments in neuroscience, researchers will need to focus on age differences in brainsystems and differences in brain regions or structures considered independently, and how brain development affects adolescent behavior.

## Conclusion

Though, age as a criterion to determine the criminal responsibility of minors has economic benefits (the distinction is clear and simple, and normal circumstances do not require a lot of legal procedures to confirm), psychological science and neuroscience tend to challenge the public view, that the relationship between the age of adolescents and the index of criminal responsibility capacity (adolescents’ dialectical thinking ability, self-control ability and empathy ability) is more complicated because of the non-linear development of the certain traits. In a word, age is not the only basis on which to judge a juvenile’s criminal responsibility. In recent years, many countries have chosen to combat juvenile delinquency by lowering the age of criminal responsibility. Not only is this measure contrary to the intent of the UN Convention on the Rights of the Child and inconsistent with adolescents’ developmental patterns; there is also clear, overwhelming evidence that exposing adolescents to the justice system too early is not conducive to their rehabilitation. Therefore, it is urgent to reflect on how to set a minimum age of criminal responsibility and balance the relationship between the punishment of juvenile crimes and the protection of victims’ rights.

### Raise the Minimum Age of Criminal Responsibility

As we found that the slow development of dialectical thinking ability in adolescents (emerging adults), and their ability to control themselves sharply during adolescence (around age 15), a more desirable compromised solution or measure would be for political states to raise the age of criminal responsibility for adolescents, rather than the current orientation toward lowering it (Fowler and Kurlychek, 2018; Hong, 2020). The benefits of raising the minimum age of criminal responsibility would be to confirm age differences in legally relevant ones (the boundary is relatively clear, saving lots of judicial review resources), and further highlight the protection of the rights of young people to develop. The approach for minors will weaken the label effect caused by their crimes, which is not only conducive to the correction of minors’ deviant behavior, but also conducive to the re-socialization of minors after education and guidance. on the contrary, the way the current political countries lower the minimum age of criminal responsibility to counter their deviant behavior is undoubtedly shirking the responsibility of the state and society, as minors are at the social stage, easily influenced by the social environment, and their deviant behavior needs more tolerance and positive guidance.

### Accept the Rebuttable Presumption of Doli Incapax

The rebuttable presumption of doli incapax, derived from ancient Roman law, needs to be taken into account. This system measures the capacity to commit a crime not so much in terms of age as in terms of the understanding and judgment of the juvenile offender (Blackstone, 1966). Faced with criminal cases, juveniles below the minimum age of criminal responsibility (we encouraged legislators firstly to set a relatively higher age of criminal responsibility) can be pursued if the prosecution can provide the court with a “very clear and complete evidence” that the accused knew what they were doing was “seriously wrong” (or presumed to have mens rea)(Van Krieken, 2013; Lennings and Lennings, 2014). This necessitates a professionally qualified person to assess young children’s capacity for criminal responsibility, including their cognitive, moral, emotional, psychological, and social growth [South African, Child Justice Act 75 of 2008, s11(2),(3)]. Doli incapax is consistent with the concept of criminal responsibility and the fact of juvenile development (Crofts, 2016); it is also in line with the basic principle of criminal law that “no penalty should be applied to a person unless he [or she] has had [the] capacity and a fair opportunity to adapt his [or her] conduct to the law” (Hart, 1968, p. 181).

In brief, we encouraged legislators to set a relatively higher age of criminal responsibility. Juveniles below this age can be pursued if the prosecution can prove they committed a crime with mens rea.

## Data Availability Statement

The original contributions presented in the study are included in the article/Supplementary Material, further inquiries can be directed to the corresponding authors.

## Author Contributions

YS: ideas, data collection, writing, and revisions. YF: data analysis, writing, and revisions. BM: data analysis and writing. LW: data analysis and revisions. DW: ideas and data collection. All authors contributed to the article and approved the submitted version.

## Conflict of Interest

The authors declare that the research was conducted in the absence of any commercial or financial relationships that could be construed as a potential conflict of interest.

## Publisher’s Note

All claims expressed in this article are solely those of the authors and do not necessarily represent those of their affiliated organizations, or those of the publisher, the editors and the reviewers. Any product that may be evaluated in this article, or claim that may be made by its manufacturer, is not guaranteed or endorsed by the publisher.
