# Supplementary material for: Psychometric Challenges in the Measurement of Constructs Underlying Criminal Responsibility in Children and Young Adults: A Cross-Sectional Study
Source: Front Psychol. 2022 Jan 13;12:781669. doi: 10.3389/fpsyg.2021.781669 (PMC8792403; doi:10.3389/fpsyg.2021.781669)
Supplement: Supplementary file 1 [file Data_Sheet_1.PDF]

Appendix A: Bonferroni Post Hoc Test of Three Abilities of Respondents of Different Ages

		Mean Difference	SE	P	95%CI	
B-DSS						
11	12	-0.02440	0.04529	1.000	-0.1693	0.1205
	13	0.00657	0.04474	1.000	-0.1366	0.1497
	14	-0.00845	0.04469	1.000	-0.1515	0.1345
	15	-0.08749	0.04472	1.000	-0.2306	0.0556
	16	-0.00068	0.04518	1.000	-0.1453	0.1439
	17	0.00462	0.04531	1.000	-0.1404	0.1496
	18	0.07358	0.04376	1.000	-0.0664	0.2136
	19	0.03800	0.05266	1.000	-0.1305	0.2065
12	11	0.02440	0.04529	1.000	-0.1205	0.1693
	13	0.03097	0.03443	1.000	-0.0792	0.1411
	14	0.01595	0.03437	1.000	-0.0940	0.1259
	15	-0.06309	0.03441	1.000	-0.1732	0.0470
	16	0.02371	0.03501	1.000	-0.0883	0.1357
	17	0.02902	0.03518	1.000	-0.0835	0.1416
	18	0.09797	0.03315	0.113	-0.0081	0.2040
	19	0.06239	0.04424	1.000	-0.0792	0.2040
13	11	-0.00657	0.04474	1.000	-0.1497	0.1366
	12	-0.03097	0.03443	1.000	-0.1411	0.0792
	14	-0.01502	0.03363	1.000	-0.1226	0.0926
	15	-0.09406	0.03367	0.189	-0.2018	0.0137
	16	-0.00725	0.03429	1.000	-0.1170	0.1025
	17	-0.00195	0.03446	1.000	-0.1122	0.1083
	18	0.06700	0.03238	1.000	-0.0366	0.1706
	19	0.03143	0.04367	1.000	-0.1083	0.1712
14	11	0.00845	0.04469	1.000	-0.1345	0.1515
	12	-0.01595	0.03437	1.000	-0.1259	0.0940

	13	0.01502	0.03363	1.000	-0.0926	0.1226
	15	-0.07904	0.03361	0.675	-0.1866	0.0285
	16	0.00777	0.03422	1.000	-0.1017	0.1173
	17	0.01307	0.03439	1.000	-0.0970	0.1231
	18	0.08203	0.03232	0.403	-0.0214	0.1854
	19	0.04645	0.04362	1.000	-0.0931	0.1860
15	11	0.08749	0.04472	1.000	-0.0556	0.2306
	12	0.06309	0.03441	1.000	-0.0470	0.1732
	13	0.09406	0.03367	0.189	-0.0137	0.2018
	14	0.07904	0.03361	0.675	-0.0285	0.1866
	16	0.08681	0.03426	0.408	-0.0228	0.1964
	17	0.09211	0.03444	0.271	-0.0181	0.2023
	18	0.16107*	0.03236	0.000	0.0575	0.2646
	19	0.12549	0.04365	0.147	-0.0142	0.2652
16	11	0.00068	0.04518	1.000	-0.1439	0.1453
	12	-0.02371	0.03501	1.000	-0.1357	0.0883
	13	0.00725	0.03429	1.000	-0.1025	0.1170
	14	-0.00777	0.03422	1.000	-0.1173	0.1017
	15	-0.08681	0.03426	0.408	-0.1964	0.0228
	17	0.00530	0.03503	1.000	-0.1068	0.1174
	18	0.07426	0.03300	0.882	-0.0313	0.1798
	19	0.03868	0.04413	1.000	-0.1025	0.1799
17	11	-0.00462	0.04531	1.000	-0.1496	0.1404
	12	-0.02902	0.03518	1.000	-0.1416	0.0835
	13	0.00195	0.03446	1.000	-0.1083	0.1122
	14	-0.01307	0.03439	1.000	-0.1231	0.0970
	15	-0.09211	0.03444	0.271	-0.2023	0.0181
	16	-0.00530	0.03503	1.000	-0.1174	0.1068
	18	0.06896	0.03317	1.000	-0.0372	0.1751

	19	0.03338	0.04426	1.000	-0.1082	0.1750
18	11	-0.07358	0.04376	1.000	-0.2136	0.0664
	12	-0.09797	0.03315	0.113	-0.2040	0.0081
	13	-0.06700	0.03238	1.000	-0.1706	0.0366
	14	-0.08203	0.03232	0.403	-0.1854	0.0214
	15	-0.16107*	0.03236	0.000	-0.2646	-0.0575
	16	-0.07426	0.03300	0.882	-0.1798	0.0313
	17	-0.06896	0.03317	1.000	-0.1751	0.0372
	19	-0.03558	0.04267	1.000	-0.1721	0.1009
19	11	-0.03800	0.05266	1.000	-0.2065	0.1305
	12	-0.06239	0.04424	1.000	-0.2040	0.0792
	13	-0.03143	0.04367	1.000	-0.1712	0.1083
	14	-0.04645	0.04362	1.000	-0.1860	0.0931
	15	-0.12549	0.04365	0.147	-0.2652	0.0142
	16	-0.03868	0.04413	1.000	-0.1799	0.1025
	17	-0.03338	0.04426	1.000	-0.1750	0.1082
	18	0.03558	0.04267	1.000	-0.1009	0.1721
SAMSSQ						
11	12	0.14593	0.04934	0.113	-0.0120	0.3038
	13	0.36449*	0.04874	0.000	0.2085	0.5204
	14	0.59970*	0.04868	0.000	0.4439	0.7555
	15	0.70273*	0.04872	0.000	0.5468	0.8586
	16	0.70271*	0.04920	0.000	0.5453	0.8601
	17	0.68783*	0.04936	0.000	0.5299	0.8458
	18	0.53906*	0.04767	0.000	0.3865	0.6916
	19	0.44386*	0.05737	0.000	0.2603	0.6274
12	11	-0.14593	0.04934	0.113	-0.3038	0.0120
	13	0.21856*	0.03751	0.000	0.0985	0.3386
	14	0.45377*	0.03744	0.000	0.3340	0.5736

	15	0.55681*	0.03749	0.000	0.4369	0.6768
	16	0.55678*	0.03811	0.000	0.4348	0.6787
	17	0.54190*	0.03832	0.000	0.4193	0.6645
	18	0.39313*	0.03611	0.000	0.2776	0.5087
	19	0.29793*	0.04819	0.000	0.1437	0.4521
13	11	-0.36449*	0.04874	0.000	-0.5204	-0.2085
	12	-0.21856*	0.03751	0.000	-0.3386	-0.0985
	14	0.23521*	0.03664	0.000	0.1180	0.3524
	15	0.33825*	0.03668	0.000	0.2209	0.4556
	16	0.33822*	0.03732	0.000	0.2188	0.4577
	17	0.32334*	0.03754	0.000	0.2032	0.4435
	18	0.17457*	0.03528	0.000	0.0617	0.2875
	19	0.07937	0.04757	1.000	-0.0729	0.2316
14	11	-0.59970*	0.04868	0.000	-0.7555	-0.4439
	12	-0.45377*	0.03744	0.000	-0.5736	-0.3340
	13	-0.23521*	0.03664	0.000	-0.3524	-0.1180
	15	0.10304	0.03661	0.177	-0.0141	0.2202
	16	0.10301	0.03725	0.206	-0.0162	0.2222
	17	0.08813	0.03747	0.674	-0.0318	0.2080
	18	-0.06064	0.03521	1.000	-0.1733	0.0520
	19	-0.15584*	0.04752	0.038	-0.3079	-0.0038
15	11	-0.70273*	0.04872	0.000	-0.8586	-0.5468
	12	-0.55681*	0.03749	0.000	-0.6768	-0.4369
	13	-0.33825*	0.03668	0.000	-0.4556	-0.2209
	14	-0.10304	0.03661	0.177	-0.2202	0.0141
	16	-0.00003	0.03730	1.000	-0.1194	0.1193
	17	-0.01490	0.03751	1.000	-0.1349	0.1051
	18	-0.16368*	0.03525	0.000	-0.2765	-0.0509
	19	-0.25888*	0.04755	0.000	-0.4110	-0.1067

16	11	-0.70271*	0.04920	0.000	-0.8601	-0.5453
	12	-0.55678*	0.03811	0.000	-0.6787	-0.4348
	13	-0.33822*	0.03732	0.000	-0.4577	-0.2188
	14	-0.10301	0.03725	0.206	-0.2222	0.0162
	15	0.00003	0.03730	1.000	-0.1193	0.1194
	17	-0.01488	0.03814	1.000	-0.1369	0.1072
	18	-0.16365*	0.03592	0.000	-0.2786	-0.0487
	19	-0.25885*	0.04805	0.000	-0.4126	-0.1051
17	11	-0.68783*	0.04936	0.000	-0.8458	-0.5299
	12	-0.54190*	0.03832	0.000	-0.6645	-0.4193
	13	-0.32334*	0.03754	0.000	-0.4435	-0.2032
	14	-0.08813	0.03747	0.674	-0.2080	0.0318
	15	0.01490	0.03751	1.000	-0.1051	0.1349
	16	0.01488	0.03814	1.000	-0.1072	0.1369
	18	-0.14877*	0.03614	0.001	-0.2644	-0.0331
	19	-0.24397*	0.04821	0.000	-0.3983	-0.0897
18	11	-0.53906*	0.04767	0.000	-0.6916	-0.3865
	12	-0.39313*	0.03611	0.000	-0.5087	-0.2776
	13	-0.17457*	0.03528	0.000	-0.2875	-0.0617
	14	0.06064	0.03521	1.000	-0.0520	0.1733
	15	0.16368*	0.03525	0.000	0.0509	0.2765
	16	0.16365*	0.03592	0.000	0.0487	0.2786
	17	0.14877*	0.03614	0.001	0.0331	0.2644
	19	-0.09520	0.04648	1.000	-0.2439	0.0535
19	11	-0.44386*	0.05737	0.000	-0.6274	-0.2603
	12	-0.29793*	0.04819	0.000	-0.4521	-0.1437
	13	-0.07937	0.04757	1.000	-0.2316	0.0729
	14	0.15584*	0.04752	0.038	0.0038	0.3079
	15	0.25888*	0.04755	0.000	0.1067	0.4110

	16	0.25885*	0.04805	0.000	0.1051	0.4126
	17	0.24397*	0.04821	0.000	0.0897	0.3983
	18	0.09520	0.04648	1.000	-0.0535	0.2439
BES						
11	12	0.08855	0.04416	1.000	-0.0528	0.2299
	13	0.05640	0.04361	1.000	-0.0832	0.1960
	14	0.05765	0.04355	1.000	-0.0817	0.1970
	15	0.01830	0.04357	1.000	-0.1211	0.1577
	16	0.03608	0.04402	1.000	-0.1048	0.1769
	17	0.00822	0.04418	1.000	-0.1331	0.1496
	18	0.00389	0.04264	1.000	-0.1325	0.1403
	19	0.02285	0.05135	1.000	-0.1415	0.1872
12	11	-0.08855	0.04416	1.000	-0.2299	0.0528
	13	-0.03215	0.03368	1.000	-0.1399	0.0756
	14	-0.03090	0.03359	1.000	-0.1384	0.0766
	15	-0.07025	0.03362	1.000	-0.1778	0.0373
	16	-0.05247	0.03420	1.000	-0.1619	0.0570
	17	-0.08033	0.03441	0.707	-0.1904	0.0298
	18	-0.08466	0.03240	0.325	-0.1883	0.0190
	19	-0.06570	0.04323	1.000	-0.2040	0.0726
13	11	-0.05640	0.04361	1.000	-0.1960	0.0832
	12	0.03215	0.03368	1.000	-0.0756	0.1399
	14	0.00124	0.03287	1.000	-0.1039	0.1064
	15	-0.03810	0.03289	1.000	-0.1434	0.0671
	16	-0.02033	0.03349	1.000	-0.1275	0.0868
	17	-0.04818	0.03370	1.000	-0.1560	0.0597
	18	-0.05251	0.03165	1.000	-0.1538	0.0488
	19	-0.03355	0.04267	1.000	-0.1701	0.1030
14	11	-0.05765	0.04355	1.000	-0.1970	0.0817

	12	0.03090	0.03359	1.000	-0.0766	0.1384
	13	-0.00124	0.03287	1.000	-0.1064	0.1039
	15	-0.03935	0.03281	1.000	-0.1443	0.0656
	16	-0.02157	0.03340	1.000	-0.1285	0.0853
	17	-0.04942	0.03362	1.000	-0.1570	0.0582
	18	-0.05375	0.03157	1.000	-0.1548	0.0473
	19	-0.03479	0.04261	1.000	-0.1711	0.1015
15	11	-0.01830	0.04357	1.000	-0.1577	0.1211
	12	0.07025	0.03362	1.000	-0.0373	0.1778
	13	0.03810	0.03289	1.000	-0.0671	0.1434
	14	0.03935	0.03281	1.000	-0.0656	0.1443
	16	0.01778	0.03342	1.000	-0.0892	0.1247
	17	-0.01008	0.03364	1.000	-0.1177	0.0976
	18	-0.01441	0.03159	1.000	-0.1155	0.0867
	19	0.00455	0.04262	1.000	-0.1318	0.1409
16	11	-0.03608	0.04402	1.000	-0.1769	0.1048
	12	0.05247	0.03420	1.000	-0.0570	0.1619
	13	0.02033	0.03349	1.000	-0.0868	0.1275
	14	0.02157	0.03340	1.000	-0.0853	0.1285
	15	-0.01778	0.03342	1.000	-0.1247	0.0892
	17	-0.02785	0.03422	1.000	-0.1374	0.0816
	18	-0.03218	0.03221	1.000	-0.1352	0.0709
	19	-0.01322	0.04308	1.000	-0.1511	0.1246
17	11	-0.00822	0.04418	1.000	-0.1496	0.1331
	12	0.08033	0.03441	0.707	-0.0298	0.1904
	13	0.04818	0.03370	1.000	-0.0597	0.1560
	14	0.04942	0.03362	1.000	-0.0582	0.1570
	15	0.01008	0.03364	1.000	-0.0976	0.1177
	16	0.02785	0.03422	1.000	-0.0816	0.1374

	18	-0.00433	0.03243	1.000	-0.1081	0.0994
	19	0.01463	0.04325	1.000	-0.1238	0.1530
18	11	-0.00389	0.04264	1.000	-0.1403	0.1325
	12	0.08466	0.03240	0.325	-0.0190	0.1883
	13	0.05251	0.03165	1.000	-0.0488	0.1538
	14	0.05375	0.03157	1.000	-0.0473	0.1548
	15	0.01441	0.03159	1.000	-0.0867	0.1155
	16	0.03218	0.03221	1.000	-0.0709	0.1352
	17	0.00433	0.03243	1.000	-0.0994	0.1081
	19	0.01896	0.04167	1.000	-0.1144	0.1523
19	11	-0.02285	0.05135	1.000	-0.1872	0.1415
	12	0.06570	0.04323	1.000	-0.0726	0.2040
	13	0.03355	0.04267	1.000	-0.1030	0.1701
	14	0.03479	0.04261	1.000	-0.1015	0.1711
	15	-0.00455	0.04262	1.000	-0.1409	0.1318
	16	0.01322	0.04308	1.000	-0.1246	0.1511
	17	-0.01463	0.04325	1.000	-0.1530	0.1238
	18	-0.01896	0.04167	1.000	-0.1523	0.1144
